# Position-reconfigurable pinning for magnetic domain wall motion

**DOI:** 10.1038/s41598-023-34040-y

**Published:** 2023-04-26

**Authors:** Taekhyeon Lee, Seyeop Jeong, Sanghoon Kim, Kab-Jin Kim

**Affiliations:** 1 Department of Physics, Korea Advanced Institute of Science and Technology, Daejeon, Republic of Korea; 2 Department of Physics and Energy Harvest Storage Research Center, Ulsan University, Ulsan, Republic of Korea

**Keywords:** Magnetic properties and materials, Spintronics

## Abstract

Precise control of magnetic domain wall (DW) motion is crucial for DW-based spintronic devices. To date, artificially designed DW pinning sites, such as notch structures, have been used to precisely control the DW position. However, the existing DW pinning methods are not reconfigurable because they cannot change the position of pinning site after being fabricated. Herein, a novel method for attaining reconfigurable DW pinning is proposed, which relies on the dipolar interactions between two DWs located in different magnetic layers. Repulsion between DWs in both layers was observed, indicating that one of the DWs acts as a pinning barrier for the other. Because the DW is mobile in the wire, the position of pinning can be modulated, thereby resulting in reconfigurable pinning that was experimentally demonstrated for current-driven DW motion. These findings provide additional controllability of DW motion, which may expand the functionality of DW-based devices to broader spintronic applications.

## Introduction

Magnetic domain walls (DWs) are essential in the development of next-generation spintronic devices, such as racetrack memory^1–3^, DW-based logics^4–7^ and neuromorphic devices^8–13^. These emerging spintronic devices operate by accurately controlling the DW position using current. Because the DW travel distance is linearly dependent on current pulse length, DWs can be moved to a desired position ideally by adjusting the current pulse length^14^. However, accurately controlling the DW position in reality is difficult because of unexpected defects in the wire or intrinsic DW inertia, which gives a non-linear relation between the DW travel distance and current pulse length^15^. To overcome these limitations, a method has been proposed to create artificial pinning sites in the wire that can pin the DW in desired position. For instance, etched notch structures have been implemented in wires to reliably control the DW position in repeated experiments^16–19^. Applying electrical gating or ion irradiation on a specific region has been used to pin the DW at a specific position^20,21^. However, these approaches require a complicated nanofabrication process, thereby making the commercialization of DW devices cumbersome. Furthermore, nanofabrication is an irreversible process; hence, the positions of pinning sites cannot be changed after they are formed, which limits the wide applicability of DW-based devices. This limitation is particularly relevant for neuromorphic applications, where programable multilevel states are needed to emulate the synaptic functions such as weight and threshold (see Supplementary Note 1 for more details). Therefore, designing fabrication-free and position-reconfigurable pinnings is crucial to achieve DW motion-based neuromorphic applications.

**Figure 1:**
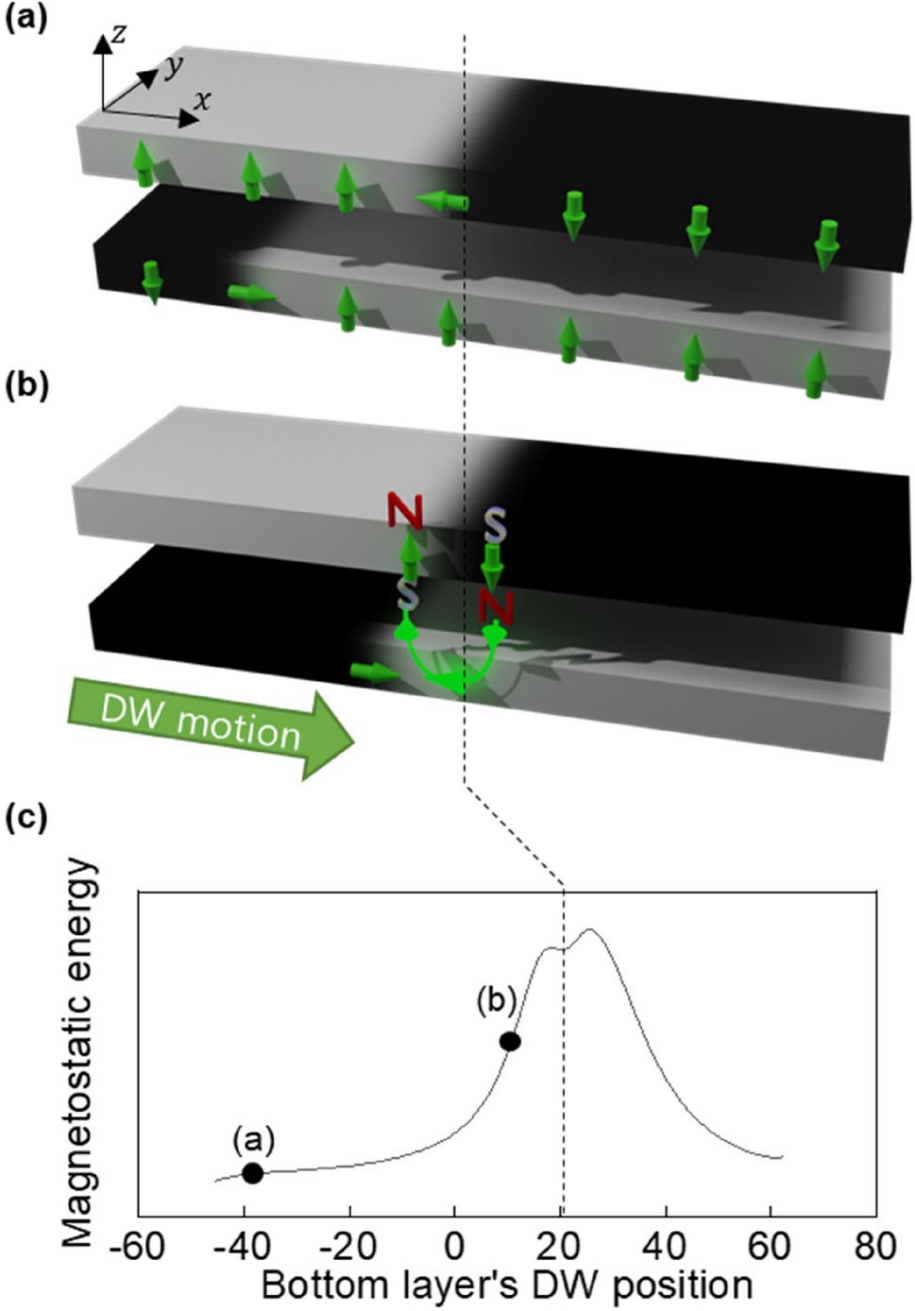
(**a**) Magnetic double layer system containing two DWs at different magnetic layers. (**b**) When the DW in bottom layer moves to the right, the DW experiences repulsive force due to the dipolar field from the top DW. (**c**) The magnetostatic energy barrier was simulated as a function of the position of the DW in the bottom layer. The dashed line denotes the position of top layer DW. The magnetostatic energies corresponding to configurations (**a** and **b**) are marked by black dots.

Herein, a novel approach for obtaining a fabrication-free and position-reconfigurable pinning site for DW motion is proposed, as illustrated schematically in Fig. 1. In contrast to previous studies requiring extrinsic treatment, the dipolar interaction of two DWs located at different magnetic layers was used. In a magnetic double layer, both DWs are chiral Néel-DWs due to inversion symmetry breaking, and thus can be moved by current through spin orbit torque^22–24^. Due to the fixed chirality, the lower layer’s DW has a magnetization configuration of “down-right-up”, resulting in the magnetization of DW center being oriented towards the (+ *x*)-direction. When this DW moves through spin–orbit torque caused by the current, the DW of the upper layer generates a dipolar field that extends to the bottom layer, oriented in the (− *x*)-direction (indicated by the green line). Therefore, the Néel type DW of bottom layer would experience a repulsive force from the dipolar field, making the top DW acts as a pinning potential barrier for the bottom DW. The energy barrier profile arising from the dipolar interaction, is calculated using micromagnetic simulation and displayed in Fig. 1c (see Supplementary Note 2 for simulation details)^25–27^.

Importantly, since the DW is a mobile structure, the position of the pinning site (i.e., the top DW’s position) can be easily modulated, resulting in reconfigurable pinning for DW motion. We note that the similar idea has been proposed theoretically using the dipolar interaction between DW and vortex^28^ but experimental demonstration has not yet been reported. To realize the proposed reconfigurable DW pinning, two conditions must be met: (1) the layer-layer interaction should be sufficiently weak, such that the two DWs can move independently, and (2) the bottom layer (driven layer) DW should be Néel-type with chirality such that it possess opposite magnetizations to the dipolar field from top layer’s DW. If not, the two DWs will attract each other (see Supplementary Note 3 for details on the attractive interaction). These conditions are satisfied by engineering the structure.

## Sample preparation and MOKE measurement

**Figure 2:**
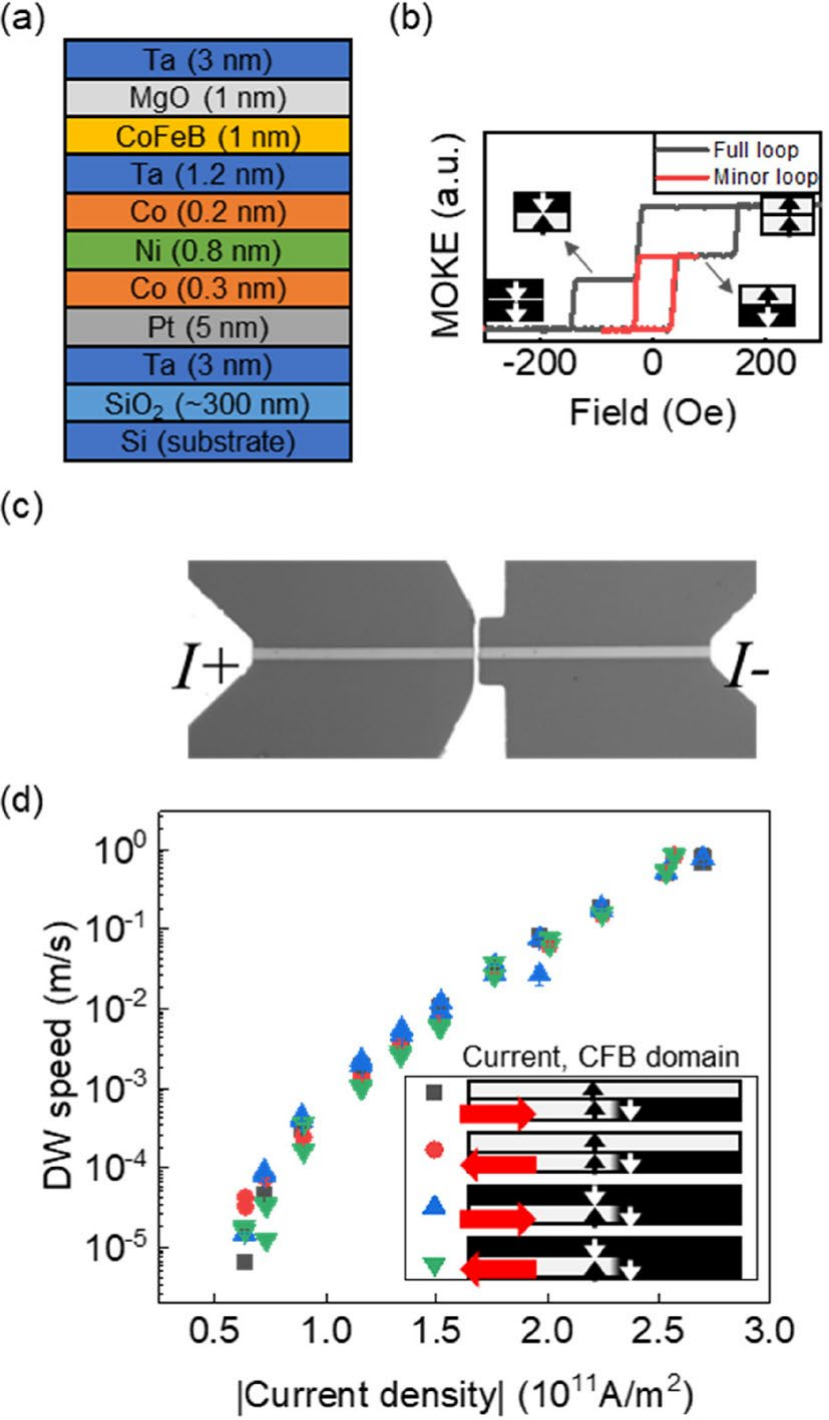
(**a**) Vertical layer structure of the proposed double magnetic layer system. (**b**) Full and minor MOKE hysteresis loops obtained in the double magnetic layer system. (**c**) Overview of wire device. (**d**) DW speed of CNC layer as a function of current density for positive and negative current while magnetization of CFB layer is saturated in either up or down (see inset for DW configuration). Direction of domain wall motion follows the current flowing direction which is denoted by red arrows in the inset.

A double magnetic layer system consisting of Si/SiO_2_/Ta(3 nm)/Pt(5 nm)/Co(0.3 nm)/Ni(0.8 nm)/Co(0.2 nm)/Ta(1.2 nm)/CoFeB(1 nm)/MgO(1 nm)/Ta(3 nm) was prepared, as shown in Fig. 2a. The top CoFeB (hereafter referred to as the CFB layer) and bottom Co/Ni/Co (hereafter referred to as the CNC layer) layers are magnetic with perpendicular magnetic anisotropy. These layers were chosen because the Pt/CNC/Ta and Ta/CFB/MgO layers have the same DW chirality owing to the same sign of the Dzyaloshinskii-Moriya interaction^29,30^. Therefore, the magnetization direction of the two DWs have a fixed chirality, as described in Fig. 1. The bottom and top Ta layers behaved as buffer and capping layers, respectively, and the Ta spacer layer in the middle was inserted to separate the two magnetic layers. The Pt layer is employed to exert spin–orbit torque on the CNC layer via the spin Hall effect^31–33^, thus enabling the verification of the proposed DW pinning experiment because the spin–orbit-torque drives the DW only in the CNC layer. Hence, the DW in the CFB layer behaves as a fixed pinning site, as described in Fig. 1b. For current-driven DW motion, the film was fabricated into a wire (width = 3 μm) using conventional photolithography and Ar ion milling, and deposited Ti/Au electrodes using the lift-off technique.

A magneto-optical Kerr effect (MOKE) microscope was used to investigate the DW motion. Figure 2b shows the MOKE intensity of the sample while sweeping the magnetic field *H* along the out-of-plane direction. A clear two-step jump is observed in the MOKE hysteresis, which indicates the individual switching of each layer. When the magnetic field increases from negative to positive, the first step is observed at approximately *H* = 20 Oe corresponding to the CFB layer switching, owing to its relatively small coercivity field compared to that of the CNC. As the magnetic field increased further, a second step appeared at approximately *H* = 150 Oe corresponding to the CNC layer switching. Particularly, the MOKE intensity for CFB is greater than that for CNC because of MOKE’s increased sensitivity near the surface owing to the finite optical penetration depth. The different MOKE intensities of CFB and CNC help to distinguish the magnetization directions of each layer, i.e., the following four different magnetization configurations can be distinguished: CFB(up)/CNC(up), CFB(up)/CNC(down), CFB(down)/CNC(up), and CFB(down)/CNC(down). The red curve in Fig. 2b indicates the minor loop of MOKE hysteresis corresponding to the reversible switching of CFB layer. The minor loop did not exhibit a sizable shift in the horizontal direction, indicating that the interlayer coupling between two magnetic layers was sufficiently weak. This means that the RKKY (Ruderman-Kittel-Kasuya-Yosida)^34–36^ coupling, which prefers antiparallel coupling of two magnetic layers, is comparable to dipolar interaction, which prefers parallel alignment between CFB and CNC layers (see Supplementary Note 4 for details). Thus, four different magnetization configurations can be stabilized at zero magnetic field, indicating that each layer’s DW can move independently.

## Result and discussion

The weak interlayer coupling was further examined by measuring the DW speed in the device shown in Fig. 2c. To this end, the current-driven DW speed in the CNC layer was measured while the magnetization of the CFB layer was saturated. The DW speed in the CNC layer is affected by the CFB layer if the interlayer coupling is strong. To verify this, the up-down DW was prepared in the CNC layer by applying external field pulse (~ ± 150 Oe, 100 ms) near the coercive field of CNC layer. Subsequently, DW speed was measured for positive and negative currents (see the Methods section for details), while the magnetization of the CFB layer was fixed in either up or down (see insets in Fig. 2d for a detailed configuration). Figure 2d shows the DW speed as a function of current density for different configurations. The results indicate that the DW speed was approximately the same for all configurations, confirming that the interlayer coupling of the CFB and CNC layers is weak. Thus, the DW in each layer can move independently.

**Figure 3:**
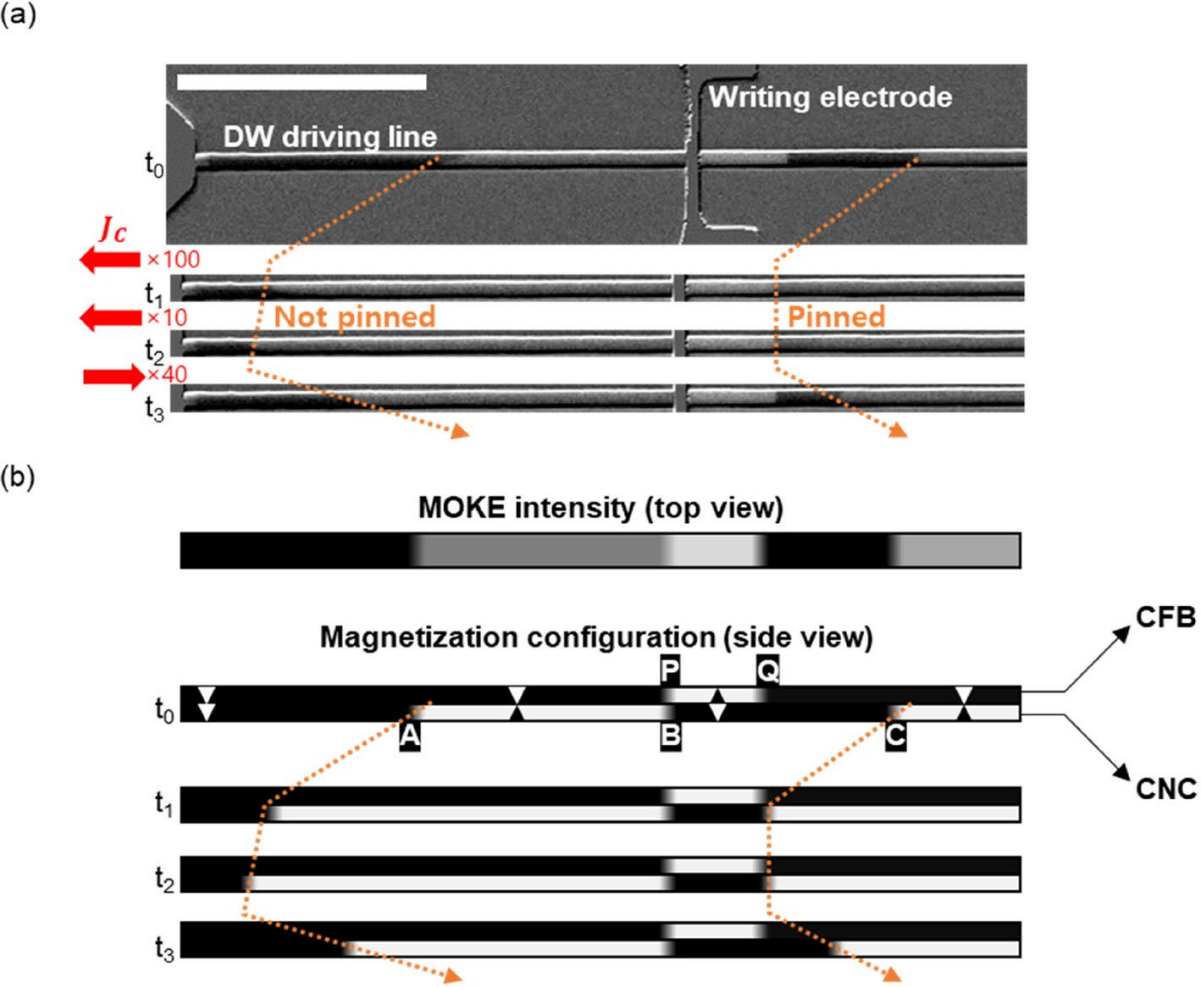
(**a**) Overall image of the patterned device and MOKE images during a series of current pulses. The scale bar is 50 μm. (**b**) Schematic illustration of magnetization configurations of each layer corresponding to the MOKE images in Fig. 3a.

The DW-DW interaction in the proposed double-layer system was investigated. To create the initial state shown in Fig. 1, a transverse writing electrode was fabricated in the middle of the wire, as shown in Fig. 3a (see Fig. 2c for full image of device). Initially, a sufficiently large *H* =  + 500 Oe was applied to saturate the magnetization of both layers, and the magnetic field was subsequently reversed to *H* =  − 150 Oe for 100 ms. For this magnetic field, the CFB layer was fully switched to the downward direction; however, the CNC layer was partially switched (see Supplementary Note 5 for details). Further, a current was injected into the transverse writing line, which generated a local Oersted field to switch the CFB layer’s magnetization near the electrode. By precisely controlling the magnetic field and current amplitude, the magnetization state was created, as shown in Fig. 3a (MOKE image) and Fig. 3b (schematic of the magnetization configuration). Notably, the magnetization configuration can be distinguished by comparing the MOKE intensities, as shown in Fig. 2b.

Figure 3b shows the three DWs (labeled A, B, and C) in the bottom CNC layer. Here, the motion of A- and C-DW located at the left and right sides of the electrode is studied because the B-DW located near the electrode hardly moves. This is because the electrode fabrication process causes local degradation beneath the electrode, which alters the magnetic anisotropy in that area. Additionally, there is a current shunting through the electrode, resulting in a significant reduction of current density near the electrode. Therefore, we focused on A- and C-DW rather than the B-DW. The CFB layer consists of two DWs (labeled P and Q), and only Q-DW is studied because the P-DW is strongly pinned at the electrode. Q-DW in the CFB layer is not moved by the current because the CFB layer is far from the bottom Pt layer. Therefore, Q-DWs in the CFB layer can act as pinning barrier when the C-DW in CNC layer approaches, which is the same situation that we described in Fig. 1b.

To check the DW-induced pinning, current pulse trains of *J* =  − 1.4 × 10^11^ A/m^2^, 50 μs were applied along the wire and the translational motion of DWs was observed (see Supplementary Note 6 for the estimation of current-induced Joule heating). As shown in Fig. 3a and b, both the A- and C-DW in the CNC layer move in the left or current-flowing direction, manifesting spin–orbit-torque-driven DW motion during $t_{0} \sim t_{1}$. The C-DW is pinned at the location of Q-DW in CFB, whereas the A-DW propagates without pinning during $t_{0} \sim t_{1}$. Thus, the Q-DW in the CFB layer acts as a pinning site for the propagation of the C-DW in the CNC layer, as proposed in Fig. 1. When the current direction was reversed in during $t_{2} \sim t_{3}$, both A-DW and C-DW propagated in right direction indicating that the C-DW is pinned because of the neighboring Q-DW instead of local defects. We note that the DW-induced pinning can work for other current densities (see Supplementary Note 7 for details).

**Figure 4:**
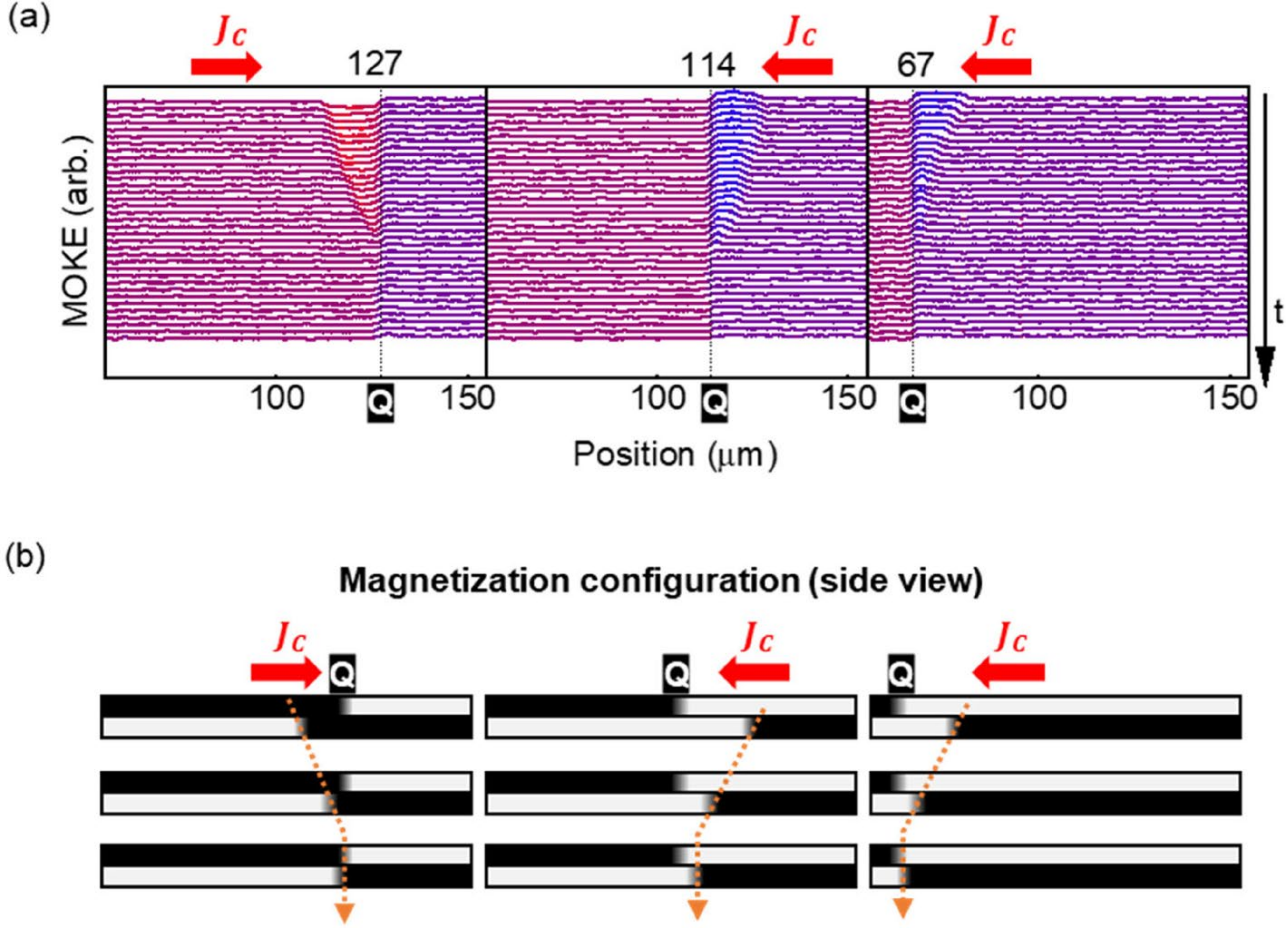
(**a**) Three experimental examples of MOKE line-profile of repeated current driven DW motion for different pinning positions and current directions. The color of points in each plot is mapped based on its MOKE intensity. The position of pinning site (Q-DW in CFB layer) is denoted by the dashed line. (**b**) Schematic illustration of magnetization configurations corresponding to Fig.4a.

To further verify the position-reconfigurability of the DW pinning site, the position of Q-DW in the CFB layer was changed and the experiments were repeated. By adjusting the initial magnetic field and writing current, three different states were prepared, as shown in the MOKE line profile in Fig. 4a where the dashed line corresponds to the Q-DW position in the CFB layer. Schematics of the magnetization configurations are shown in Fig. 4b. A current pulse of *J* =  ± 1.3 × 10^11^ A/m^2^, 100 μs was applied between the line-profile measurements. The results indicate that the DW in the CNC layer was pinned at the location of Q-DW in the CFB layer, irrespective of propagation direction. Therefore, the position of DW pinning site can be changed by varying the DW’s position without an additional fabrication process. Herein, the position of Q-DW in the CFB layer was controlled using the magnetic field and current; however, it may be controlled using fully electrical means, for instance, by designing an additional spin-injection layer adjacent to the CFB layer. Therefore, these findings have implications in further studies on magnetic double-layer systems to improve the functionality of DW devices.

## Conclusions

In summary, a position-reconfigurable DW pinning method utilizing the DW-DW interaction in a double magnetic layer system was proposed. The first magnetic layer’s DW acts as a pinning site for the second layer’s DW owing to the dipolar interaction. Because the DW is mobile in the wire, the position of pinning site created by the DW can be easily changed without using any fabrication process. The position-reconfigurable pinning for DW motion in current-driven DW motion was experimentally demonstrated. The proposed method could provide programmable pinning for DW motion, which is essential for DW-based artificial synaptic devices requiring programmable multilevel or weight factors. Hence, this study provides additional functionality for DW-based devices, which may aid in the development of DW-based neuromorphic applications.

## Methods

### Sample preparation

Magnetic films were deposited by magnetron sputtering at base pressure 5 × 10^−8^ torr and working pressure 3 mtorr. DC magnetron sputtering power was 25W (Ta, Co, Ni) and 50W (CoFeB). In case of MgO, 50W RF power was used. Magnetic films were fabricated to the wire using conventional photolithography and Ar ion etching process. Ti(5 nm)/Au(100 nm) electrodes were also fabricated using photolithography and magnetron sputtering.

### Magnetic measurements

Magneto-optical Kerr effect (MOKE) was used to observe MOKE hysteresis loop and the motion of domain walls using homemade MOKE setup. Electric current pulses were applied by pulse generator (AV-1010-B). A displacement of DW in each MOKE snapshots between current pulse injections was measured. From the linear fitting of DW displacement versus the accumulated pulse width (= time), we were able to determine the speed of DW in current-induced DW motion.

## Supplementary Information

Supplementary Information.
