# Supplementary material for: Position-reconfigurable pinning for magnetic domain wall motion
Source: Sci Rep. 2023 Apr 26;13:6791. doi: 10.1038/s41598-023-34040-y (PMC10133296; doi:10.1038/s41598-023-34040-y)
Supplement: Supplementary file 1 — Supplementary Information. [file 41598_2023_34040_MOESM1_ESM.docx]

Supplementary Note for

“Position-reconfigurable pinning for magnetic domain wall motion”

Taekhyeon Lee^1^, Seyeop Jeong^2^, Sanghoon Kim^2^, Kab-Jin Kim^1a)^

*^1^ Department of Physics, Korea Advanced Institute Science and Technology, Daejeon, Republic of Korea*

*^2^ Department of Physics and Energy Harvest Storage Research Center, Ulsan University, Ulsan, Republic of Korea*

**-Contents-**

**Note 1. Possible neuromorphic applications utilizing the proposed method.**

**Note 2. Micromagnetic simulation for DW motion in magnetic double layer**

**Note 3. Attractive interaction between two DWs at different magnetic layers**

**Note 4. Further verification of RKKY and dipolar coupling**

**Note 5. Initialization of domain wall by magnetic field pulse.**

**Note 6. Current-induced Joule heating**

**Note 7. Current density dependence of DW pinning and energy barrier determination**

**Note 1. Possible neuromorphic applications utilizing the proposed method.**

Neuromorphic devices generally utilize weighted sum of signals from synapses^37^. Conventional on-off device can only express the binary digits, ‘0’ or ‘1’ (Fig. S1 (a)), and thus cannot be used for neuromorphic application because they cannot express the multilevel states between ‘0’ and ‘1’. However, our device can provide multilevel states by controlling the position of upper DW (Fig. S1(c)). Specifically, when the binary input “0” or ‘1’is injected, the DW in the lower layer either stops at the initial position or moves up to the position where the upper DW exists. Therefore, the output can take any value between “0” and “1” depending on the position of upper DW.

Our device has also potential benefits over conventional DW motion-based neuromorphic devices. While conventional DW motion-based devices can provide continuous change of state between ‘0’ and ‘1’ (Fig. S1(b))^38,39^, it can be challenging to incorporate configurable thresholds into each device. In contrast, our device can provide a configurable threshold by controlling the position of DW, as the maximum displacement of the lower DW is limited by the position of the upper DW. Therefore, we believe that the proposed method could be advantageous for neuromorphic applications.


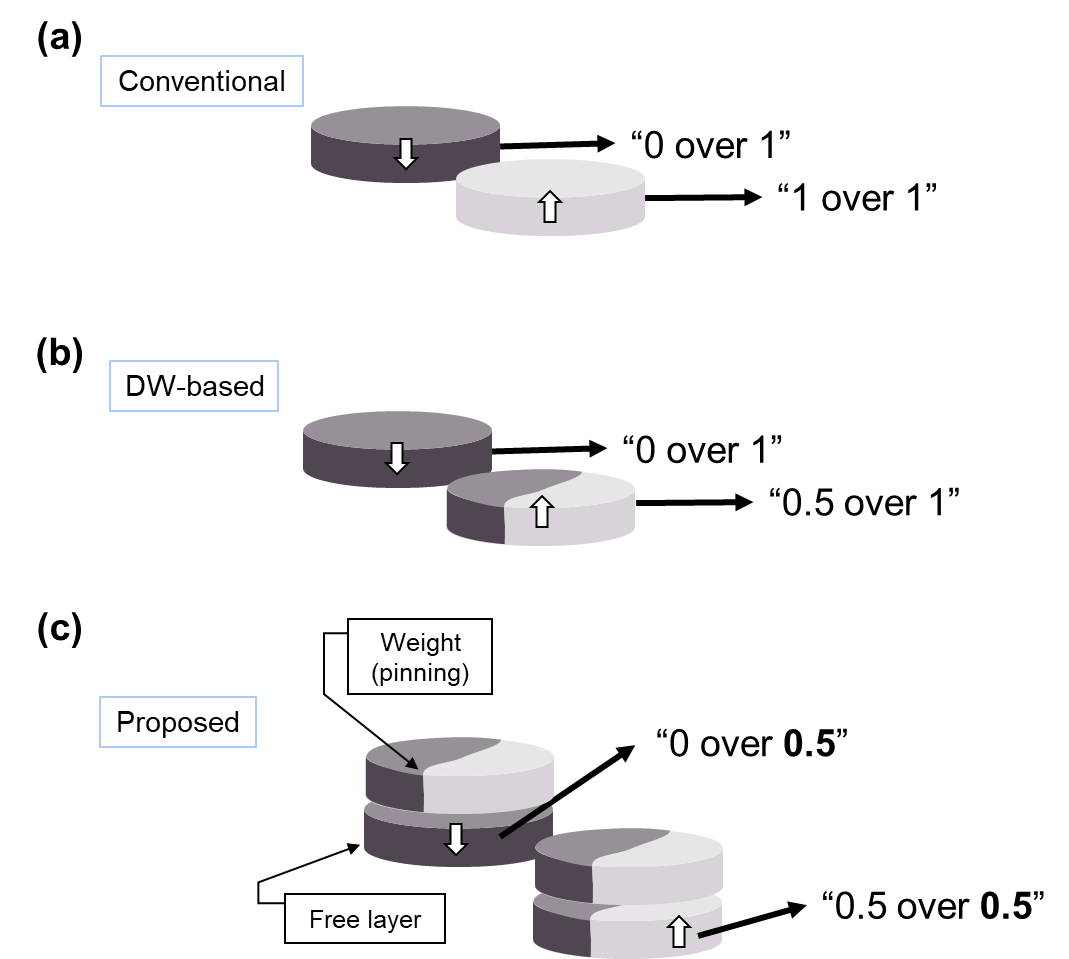


Figure. S1. (a) Conventional on/off switch with perpendicular magnet. (b) DW-based multilevel device. (c) A proposed device in which the configurable pinning can control weight/threshold for switching device.

**Note 2. Micromagnetic simulation for DW motion in magnetic double layer**

We performed micromagnetic simulation to check the DW-induced pinning in magnetic double layer. The simulation was carried out by using Mumax^3^ with the device geometry illustrated in Fig. S2. Material parameters are chosen based on the experimentally determined values. The initial position of the upper layer’s DW was set to +20 nm, while the lower layer’s DW was set to -50 nm. Figure S3 exhibits the temporal evolution of DW position when the DW moves by current. It is evident that the lower layer’s DW is pinned at the position of upper layer’s DW, suggesting the presence of DW-DW interaction.


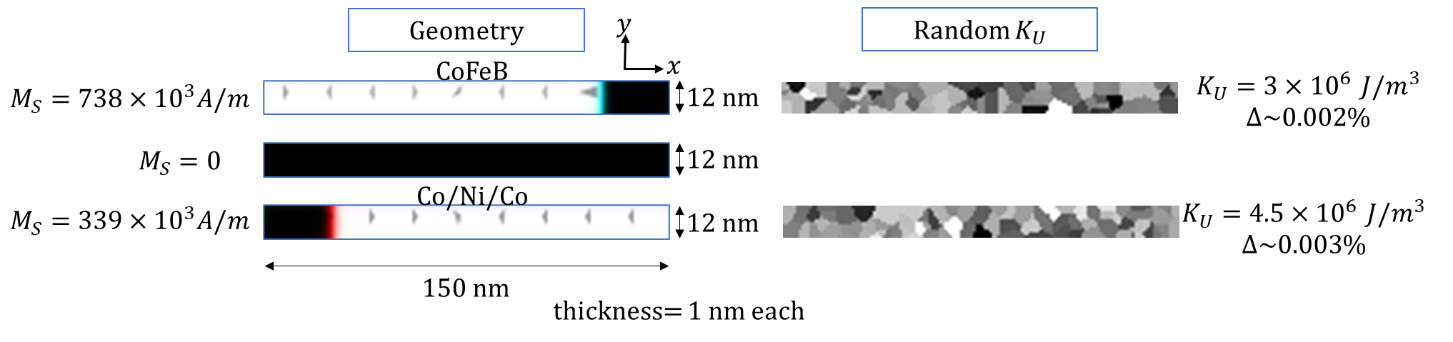


Figure. S2. Geometry used in micromagnetic simulation. Wire length and width were 150 nm and 12 nm, respectively. Thickness of CFB, Ta spacer, and CNC were set to be 1 nm with cell size of 1 nm **×** 1 nm **×**1 nm. Random anisotropy inside the wire was considered to mimic real devices.


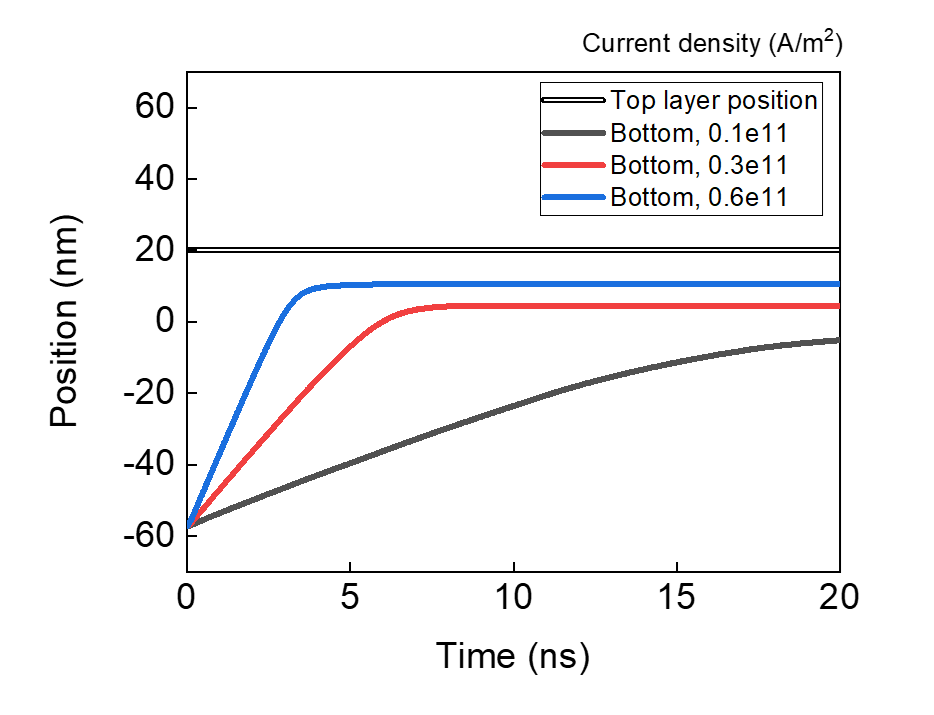


Figure. S3. The DW position during current driven DW motion with various current densities.

**Note 3. Attractive interaction between two DWs at different magnetic layers**

To achieve the attractive dipolar interaction, the direction of DW magnetization in the lower layer can be switched by reversing the sign of DMI, which determines the DW chirality. The sign of DMI is primarily determined by symmetry breaking at the interface and can be adjusted by selecting appropriate materials and engineering the interface accordingly.

It is important to note, however, that the attractive dipolar interaction is not ideal for applications because it generates an energy dip, which can trap the DW, rather than an energy barrier, which can repel the DW. While the energy dip can also pin the DW, it is much more difficult for the DW to escape from the dip. As a result, the attractive dipolar interaction is not the best option for applications that require bi-directional mobility.

To evaluate the effect of the attractive dipolar interaction, we performed simulations by reversing the DMI of the lower layer. This reversal results in the DW experiencing attractive dipolar interaction. The simulation results are presented in Fig. S4, which illustrates that the lower layer’s DW is also pinned at the position of the upper layer’s DW, even when the dipolar interaction is attractive. However, when we changed the direction of the current at t = 20 ns, the DW could not escape from the pinning in the case of attractive dipolar interaction. In contrast, the DW could easily move backward in the case of repulsive dipolar interaction. Therefore, the repulsive dipolar interaction, which we experimentally demonstrated in our work, is more suitable for applications.


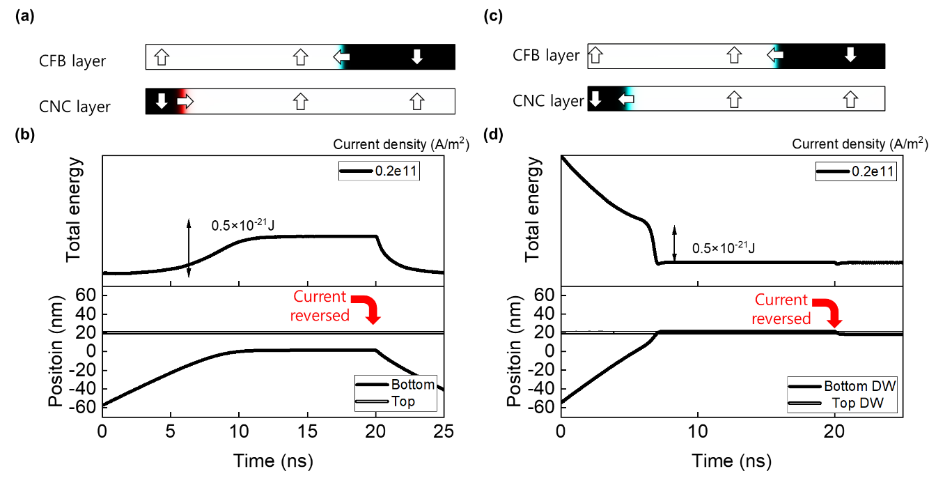


Figure. S4. (a) Micromagnetic simulation configuration corresponding to the sample in manuscript, and (b) Magnetostatic energy (top panel) and DW’s position (bottom panel) as a function of time. (c), (d) Simulation results in the same situation as Figs. R16(a) and (b) but opposite chirality of bottom layer DW.

**Note 4. Further verification of RKKY and dipolar coupling**

The presence of RKKY coupling in a magnetic double-layer system is revealed through the minor hysteresis loop, which involves switching of only one magnetic layer. When we switch only one magnetic layer, the coercive field may vary depending on the magnetization of the other layer because there are interactions between two layers such as dipolar and RKKY interactions (see Fig. S5(a)). This leads to a horizontal shift in the minor hysteresis curve. Since the dipolar interaction favours parallel alignment, while the RKKY interaction stabilizes the antiparallel alignment, the direction of loop shift can be opposite for each of these interactions. However, if the strength of two interactions is similar, the loop shift can be negligible.

As shown in Fig. S5(b), we observed that the minor loop shift is negligible in our sample. This implies that the RKKY coupling compensates the dipolar coupling. To further clarify the existence of RKKY coupling, we performed additional experiment. To this end, we prepared another sample with a slightly different stack thickness, resulting in a stronger strength of RKKY coupling from what we used in the manuscript. The red curve in Fig. S5(b) shows that the minor loop of new trial sample is clearly shifted to the negative field direction. This means that the antiparallel alignment remains stable even at zero field, indicating the presence of RKKY coupling that dominates over the dipolar coupling.


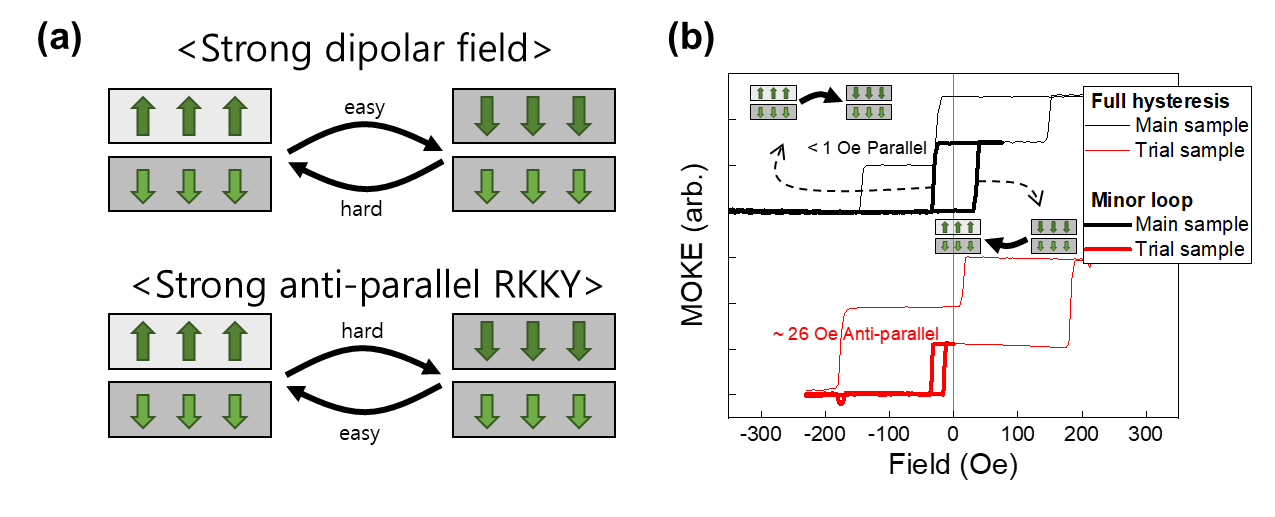


Figure. S5. (a) Magnetization switching of upper layer in the presence of the strong dipolar coupling (upper) or in the presence of the strong RKKY coupling (lower). (b) Full and minor hysteresis loop of double magnetic layers of the original sample shown in manuscript (black) and newly fabricated trial sample (red). Note that trial sample shows a strong antiparallel RKKY coupling, resulting in a minor loop being shifted to the left.

**Note 5. Initialization of domain wall by magnetic field pulse.**

As illustrated in Fig. 2(b) of the revised manuscript, the coercive field of the CFB layer is approximately 20 Oe, while that of the CNC layer is approximately 150 Oe. Therefore, applying a magnetic field of ±150 Oe for a sufficiently long time will fully switch both the CFB and CNC layers. However, in our experiment, we applied magnetic field pulse of only 100 ms duration, which was not long enough to fully switch the CNC layer. Consequently, a part of CNC layer was not switched, resulting in the formation of a domain wall.

To clarify this, we conducted additional experiments. Figure S6 presents the MOKE profile graph for various magnetic field strengths (with a fixed field duration of 100 ms). The black line corresponds to the up-saturated state after applying a + 300 Oe external field, while the other colored lines correspond to magnetic fields of -168 Oe ~ - 126 Oe. The magnetization configuration for each layer corresponding the MOKE profile is marked on the y-axis (CFB/CNC), and the schematic diagram of the magnetization state for *H* = -168 Oe and -133 Oe is also shown at the bottom of Fig. S6. Note that a larger portion of the CNC layer was reversed as the magnetic field became stronger. This approach allowed us to initialize the initial position of the domain wall in the CNC (bottom) layer.


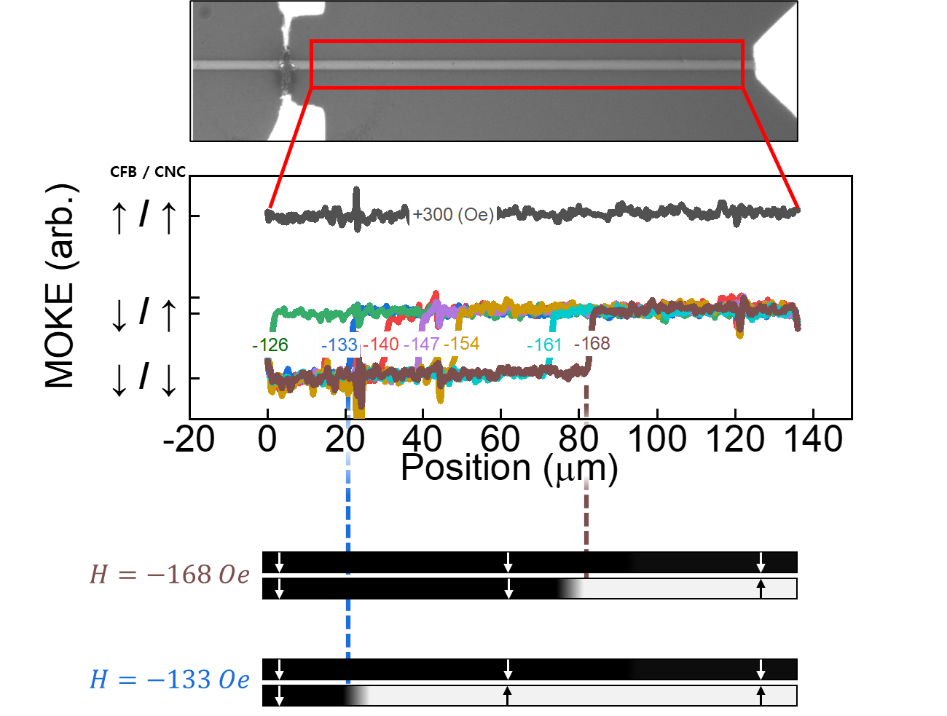


Figure. S6. MOKE profile along wire after initialization process with various negative field strengths.

**Note 6. Current-induced Joule heating**

We carried out additional experiment to measure temperature increasement during current pulse injection. First, we placed our sample on the ambient heater stage and measured sample resistance from room temperature to 70 degrees Celsius. Temperature dependence of resistance was found to be approximately 0.078 %/K, as shown in Fig. S7 (a). We then used real-time measurement, as described in Ref. 40, to estimate the resistance variation caused by current pulse (100 μs). Figure S7(b) shows that within the current density range used in manuscript (1.3~1.4×10^11^ A/m^2^), sample temperature would increase by less than 20 degrees. While this temperature increase may affect DW-induced pinning since the depinning energy barrier depends on the temperature, we note that the proposed DW pinning method can still work effectively, as the energy barrier is sufficiently high in our device (refer to Supplementary Note 7).


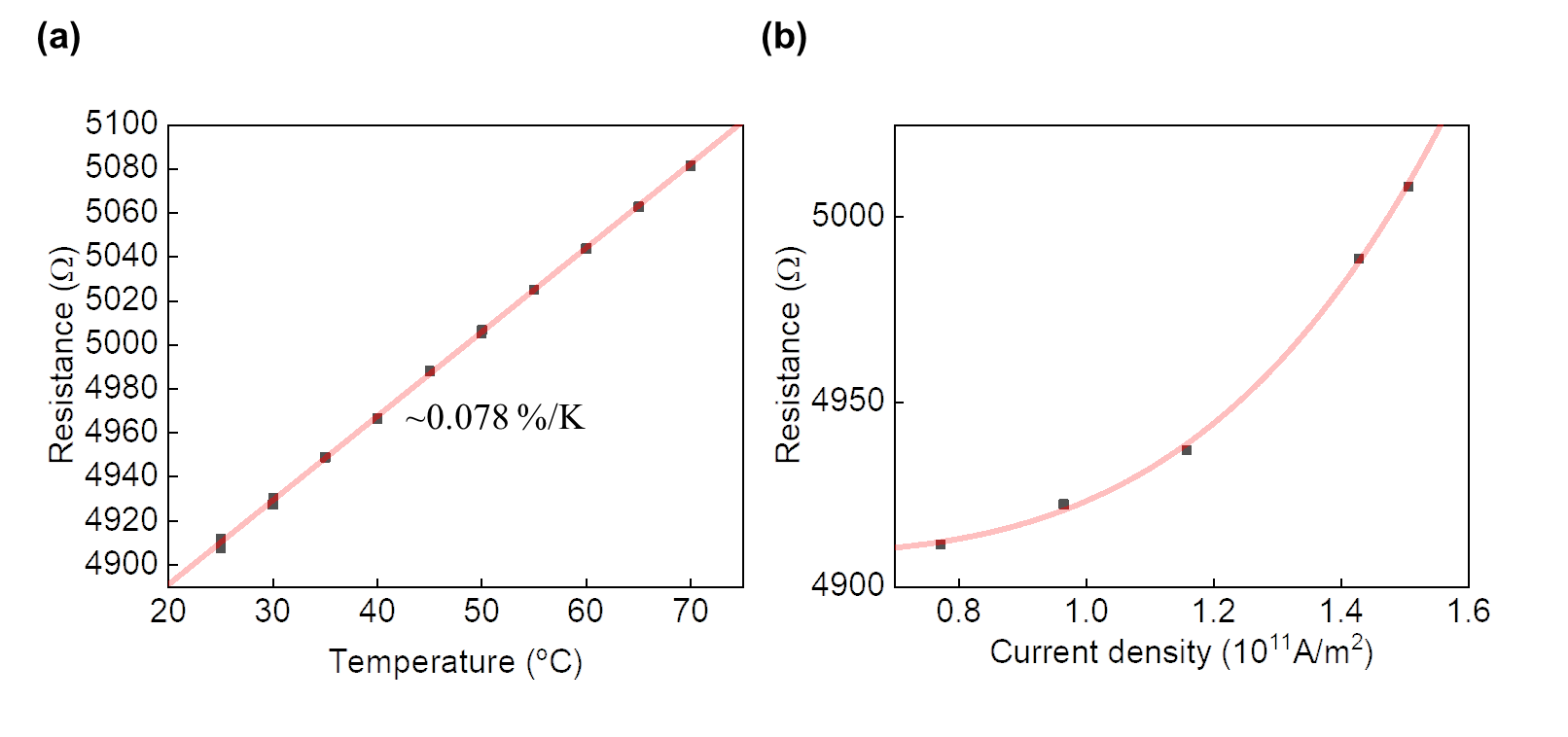


Figure. S7. (a) DC electrical resistance measured at various stage temperature. (b) The electrical resistance measured during a 100 μs current pulse application.

**Note 7. Current density dependence of DW pinning and energy barrier determination**

As the pinning energy barrier depends on the current density, we performed additional experiment to determine the energy barrier of DW-induced pinning. Following the method in Ref. 41, we measured the DW depinning time as a function of current density in thermally activated regime, as shown in Fig. S8. Based on the Arrhenius law, the pinning energy barrier is estimated to be ~ 42 k_B_T which is sufficiently large for memory or neuromorphic application. Notably, the strength of the energy barrier is determined by the dipolar field, which means that it can be further improved through layer structure engineering. We lastly remark that the pinning energy barrier can be different depending on whether the DW is in a static or dynamic state^42^. While the difference between the static and dynamic pinning could be interesting in our DW-induced pinning mechanism, we believe that it falls beyond the scope of this work.


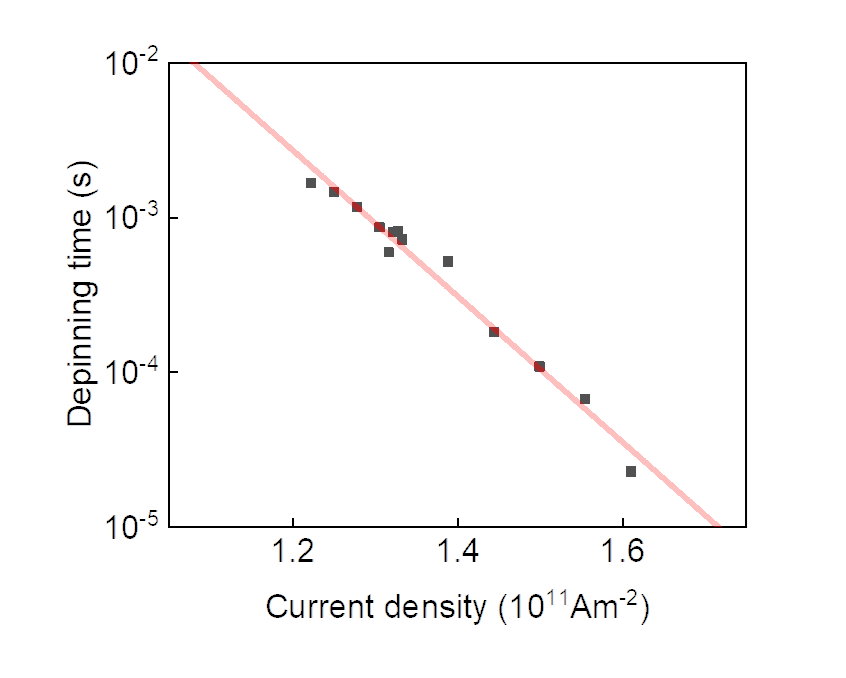


Figure. S8. Depinning time versus current density. Red line represents the fitting line following the Arrhenius law.

**References**

37. S. Jung *et al., Nature*, 601, 211-216 (2022).

38. Yang, S. *et al.* *NPG Asia Mater.* 13, 11 (2021).

39. Lequeux, S. *et al. Sci. Rep.* 6, 31510 (2016).

40. Kim, K.-J. *et al. Appl. Phys. Lett.* 92, 192509 (2008).

41. Bedau, D. *et al. Appl. Phys. Lett.* 97, 262502 (2010).

42. Ahn, S.-M. *et al. Appl. Phys. Lett.* 95, 152506 (2009).
